# Acute subdural haemorrhage in the postpartum period as a rare manifestation of possible HELLP (haemolysis, elevated liver enzymes, and low-platelet count) syndrome: a case report

**DOI:** 10.1186/1756-0500-7-408

**Published:** 2014-06-28

**Authors:** Malitha Patabendige

**Affiliations:** 1 University Obstetrics Unit, De Soysa Hospital for Women, Colombo, Sri Lanka

**Keywords:** Intracranial haemorrhage, Thrombocytopaenia, HELLP Syndrome, Pregnancy, Postpartum period

## Abstract

**Background:**

The HELLP syndrome (haemolysis, elevated liver enzymes, and low-platelet count) occurs in about 0.5 to 0.9% of all pregnancies. With occurrence of thrombocytopaenia, it signals for several potentially lethal conditions such as complete or partial HELLP syndrome, thrombotic thrombocytopaenic purpura and acute fatty liver of pregnancy.

**Case presentation:**

A previously healthy 27-year-old, Sinhala ethnic primigravida with pregnancy-induced hypertension was admitted at 38 weeks of gestation with lower abdominal pain and a blood pressure of 140/90 mmHg. She underwent emergency Caesarian section due to faetal distress giving birth to a healthy baby girl. Since postpartum day one, she was having intermittent fever spikes. All the routine investigations were normal in the first three weeks. Platelet count started dropping from post-partum day-20 onwards. On day-23, she had developed a seizure and computed tomography scan brain showed a subdural haemorrhage. She had a platelet count of 22,000 × 10^9^/liter and was managed conservatively. She also had elevated liver enzymes, lactate dehydrogenase and bilirubin levels. Blood picture on day-24 showed haemolytic anemia. On day- 36, patient again developed seizures and she was having intermittent fever with generalized headache and signs of meningism. Computed tomography scan revealed an acute on chronic subdural haemorrhage.

**Conclusions:**

Hypertensive disorders in pregnancy should be managed as high-risk throughout the postpartum period. Development of thrombocytopaenia can be considered as an early warning sign for HELLP, thrombotic thrombocytopaenic purpura or acute fatty liver of pregnancy which are lethal conditions. Prompt recognition of intracranial haemorrhages and early neurosurgical intervention is lifesaving.

## Background

The HELLP syndrome (haemolysis, elevated liver enzymes, and low-platelet count) occurs in about 0.5 to 0.9% of all pregnancies and in 10 to 20% of cases with severe pre-eclampsia [1]. In about 70% of cases, the HELLP syndrome develops before delivery [2]. It has also been reported in the postpartum period [3]. This syndrome is more common than eclampsia and leads to devastating neurological consequences such as intracerebral haemorrhage [4]. With occurrence of thrombocytopaenia, it signals for several potentially lethal conditions such as HELLP syndrome, acute fatty liver of pregnancy (AFLP) and thrombotic thrombocytopaenic purpura (TTP). Fever might be an associated symptom or an unrelated entity. This clinical vignette demonstrates a case of controversial postpartum presentation of a patient.

## Case presentation

A previously healthy 27-year-old, Sinhala ethnic woman in her first pregnancy which was complicated with pregnancy-induced hypertension, on oral nifedipine was admitted to a provincial hospital at 38 weeks of gestation. She had complained of lower abdominal pain and a blood pressure of 140/90 mmHg. She underwent an emergency Caesarian section due to faetal distress with suspicion of placental abruption on clinical grounds and giving a birth to a healthy baby girl at the provincial hospital. Caesarian section was performed with subarachnoid spinal anaesthesia under strict aseptic conditions after infiltration of the local skin with 2% lignocaine 2 ml at 3rd/4th lumbar vertebral disc space by a 25G pencil tip bevel spinal needle with 5% heavy bupivacaine 2.3 ml and 15 mg fentanyl after which free flow of cerebrospinal fluid was noted. Before Caesarian section intravenous metoclopramide 10 mg and intravenous ranitidine 50 mg stat doses were given as premedication to prevent Mendelson syndrome. During the early postpartum period she was apparently well and blood pressure was 130/90 mmHg without any other abnormal clinical signs. Her routine haematological investigations done in the early postpartum period were normal.

The patient was transferred to our postnatal ward, University Obstetrics Unit, De Soysa Hospital for Women (Teaching), Colombo, Sri Lanka on postpartum (PP) day-36 for further management of the scenario discussed below.

**Figure 1:**
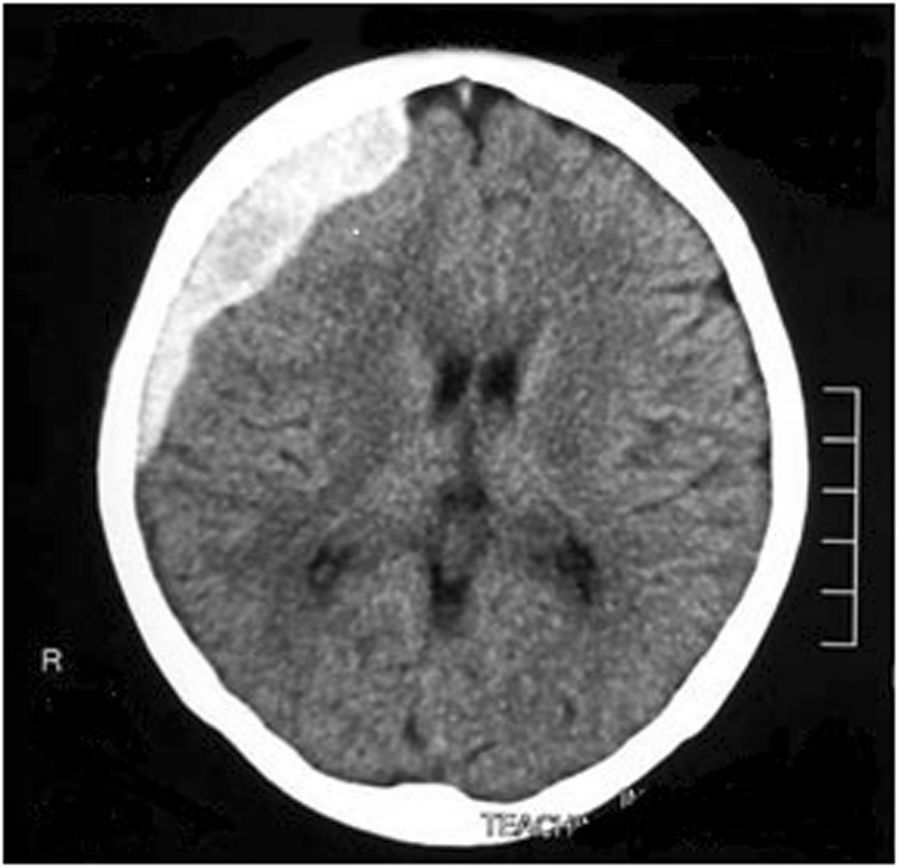
Image of computed tomography scan of brain on postpartum day 23: this shows a right-sided fronto-parietal subdural haemorrhage with midline shift.

**Figure 2:**
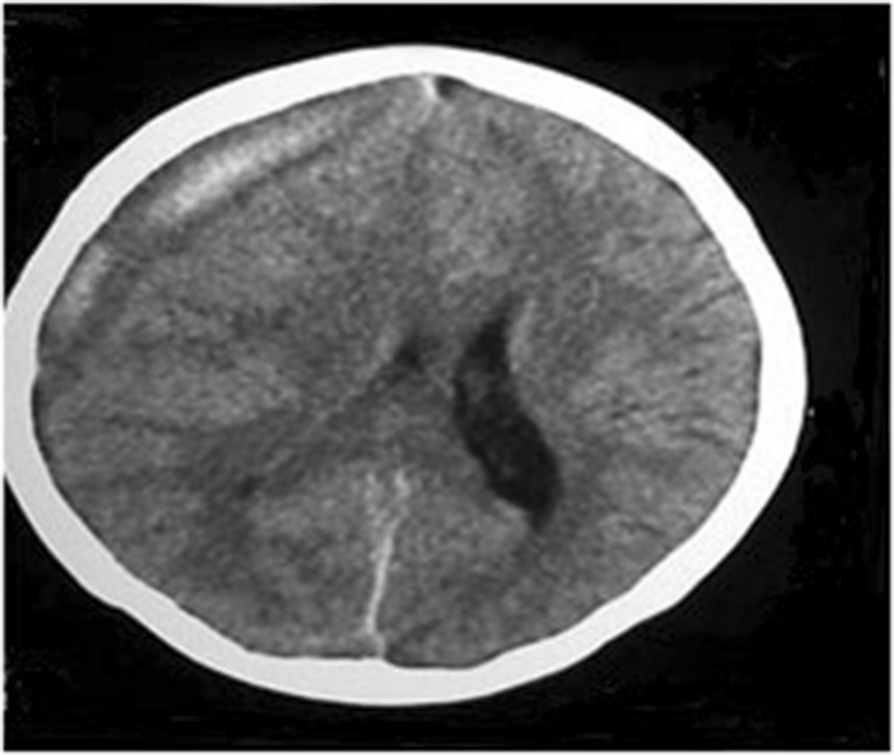
Image of computed tomography scan of brain on postpartum day 36: a right-sided acute on chronic subdural haemorrhage with midline shift and evidence of cerebral oedema.

Since PP day-01, she had had on and off fever spikes. Fever continued to occur despite intravenous antibiotics and her blood culture and urine cultures were all negative. Since PP day-01 she had been feeding the baby well and she had not any clinically significant symptoms or signs of sepsis. During this entire period she was in the provincial hospital and routine haematological and other investigations were regularly performed. Investigations done in the first 3 weeks of her postpartum period did not show any evidence of infection. Until her first presentation of seizure attack she had not had any headache or visual disturbances, only the pyrexia of unknown origin continued. Due to this continued fever she was on several intravenous antibiotics (i.e. clindamycin, ceftriaxone and carbapenum) until PP day-36 and antibiotics were omitted after emergency burr-hole aspiration of subdural haemorrhage (SDH) by the neurosurgical team. Meanwhile her 2D-echocardiography, ultrasound scan of the abdomen, computed tomography (CT) scans of chest, abdomen and pelvis done during the period of PP day-01 to day-20, were reported as normal. Since routine haematological investigations were done regularly, the decline in the platelet count was noted from PP day-20 onwards. She had continued to complain of a mild headache at that time. On PP day-23, she had developed a generalized tonic-clonic seizure and CT scan of the brain (Figure 1) showed a right-sided fronto-parietal SDH with midline shift. With a platelet count of 22,000 × 10^9^/L the SDH had been managed conservatively. Her other investigations on PP day-23 revealed as haemoglobin- 8.8 g/dL aspartate transaminase level-286 U/L, alanine transaminase level-210 U/L and lactate dehydrogenase level- 1311 U/L. Her renal function tests, serum electrolytes and clotting profile were normal. Anti-nuclear antibodies were negative. Serum total bilirubin level was 218 μmol/L with indirect bilirubin of 205 μmol/L. Blood picture done on PP day-24 had shown good evidence of haemolytic anemia, but red cell fragmentation was not obvious.She was started on prophylactic anticonvulsants. With this background, patient was having continuous on and off fever for which no other causes could be found. On PP day- 36, patient again developed a generalized tonic-clonic seizure followed by a focal seizure and transferred to a tertiary care center, University Obstetrics Unit, De Soysa Hospital for Women, Colombo, Sri Lanka for further management. On admission she was having intermittent fever spikes associated with generalized headache. Glasgow Coma Scale was 15/15 and both pupils were equal and reactive to light. Signs of meningism were seen. Blood pressure was 110/70 mmHg with hypertonia and hyper-reflexia in both lower limbs. Left-sided Babinski sign was also positive. A CT scan done on the same day (Figure 2) revealed a right-sided acute on chronic SDH with midline shift and evidence of cerebral oedema.

**Figure 3:**
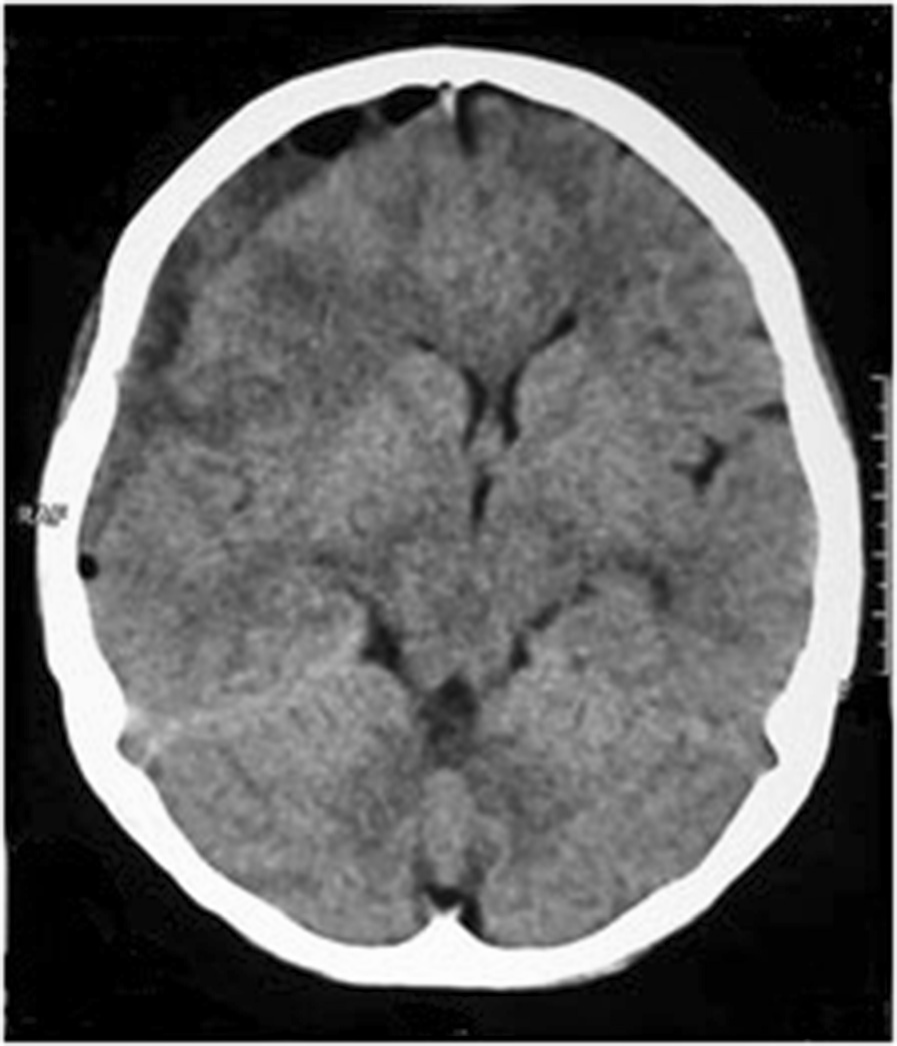
Image of computed tomography scan of brain on postpartum day 39: after the burr-hole aspiration of subdural haemorrhage.

Her platelet count was 650,000 × 10^9^/L and she underwent emergency burr-hole aspiration of SDH by the neurosurgical team removing 45 ml of blood. Repeat CT scan (Figure 3) after the aspiration showed a marked improvement with quick clinical resolution of her clinical symptoms and signs.

## Conclusions

In this case differential diagnosis includes HELLP syndrome, AFLP and TTP [3,5]. AFLP is very rare and has very poor prognosis with an estimated maternal mortality of 12.5%–18% [6]. The clinical signs of AFLP usually vary and there is significant overlapping in clinical and biochemical features with HELLP syndrome [7]. Ultrasound scan of the liver may reveal increased echogenicity in severe cases of AFLP [8]. CT scan will show decreased or diffuse attenuation in the liver [8]. This condition occurs more commonly in primigravida, twin pregnancy and pregnancies carrying a male faetus [9]. But in our case none of these were seen. The three main abnormalities found in HELLP syndrome are haemolysis, low platelet count and elevated liver enzymes. The platelet count is considered as the best indicator of HELLP syndrome followed by elevated serum lactate dehydrogenase level. In thrombotic thrombocytopaenic purpura, there can be associated renal impairment. But in this case renal functions remained normal. Therefore, this can be most likely some form of HELLP syndrome. But both AFLP and HELLP syndrome might have overlapped in this case scenario. Differentiation between TTP and HELLP syndrome is often difficult. Coexistence of TTP and HELLP is also possible [10]. In literature, it’s reported that HELLP syndrome has resulted in a fatal intracranial haemorrhage during the perinatal period of a primigravida in Japan in 2009 [11]. There was another case of HELLP syndrome with disseminated intravascular coagulation resulting in a lethal pontine haemorrhage followed by maternal death [4]. Hypertensive disorders in pregnancy are major causes of intracerebral haemorrhage during pregnancy and the puerperium [12,13].

Some pregnant women develop just one or two of the characteristics of this syndrome, which is termed as partial HELLP Syndrome (pHELLP) [14]. Complete HELLP syndrome (cHELLP) is defined by the presence of all of the three laboratory criteria according to the Tennessee Classification System [15-17]. Partial HELLP syndrome is defined by the presence of one or two features of HELLP, but not the complete form [14,15]. However a recent study has shown that partial and complete HELLP syndromes are not two distinct entities. They probably represent a continuum in the natural evolution of the same disease [15].

There is an entity called drug fever which can be induced by parenteral administration of antibiotics [18,19] and this might have been a cause for the continuation of intermittent fever in this scenario. In this patient on PP day-36 after discontinuation of the antibiotics and burr-hole aspiration of SDH, fever completely settled. It is well known that drug fever rapidly reverses after discontinuation of the offending agent [19]. It is a febrile response that appears temporarily with the administration of a particular drug and disappears after discontinuation of the drug [19]. Pregnant mothers who receive epidural analgesia are more likely to experience hyperthermia and overt clinical fever is well established [20]. But other methods like subarachnoid spinal anaesthesia as in this patient have not been reported with a significant association of occurrence of fever. Fever can occur frequently in some intracerebral haemorrhages specially after subarachnoid haemorrhage [21,22] and fever affects approximately 70% of patients with subarachnoid haemorrhage [23]. But subdural haemorrhage leading to fever was not frequently reported in literature unless it’s an infected haematoma associated with meningitis [24]. However it is more common in elderly patients [24]. In our case, there might have been an underlying small focus of meningitis which we could not confirm. Lumbar puncture for cerebrospinal fluid analysis had not been done to exclude meningitis.

Although this case scenario is a more complex one, it also has some important messages which might be useful in the postnatal care of pregnant mothers. Patients with pregnancy-induced hypertension and HELLP syndrome should be managed as high-risk throughout the postpartum period (up to six weeks). It requires regular review and involvement of a multidisciplinary team. Development of thrombocytopaenia should be considered as a sign of alarm in postpartum mothers because it can be one of the many lethal conditions causing maternal death such as HELLP, TTP or AFLP etc. Prompt recognition of thrombocytopaenia plays a key role and it signals that patient may have a very lethal condition ahead. Early detection of intracranial haemorrhages and early neurosurgical intervention is also important in saving the life of the mother.

## Consent

Written informed consent was obtained from the patient for publication of this Case Report and any accompanying images. A copy of the written consent is available for review by the Editor-in-Chief of this journal.

## Abbreviations

PP: Postpartum period; CT: Computed tomography; SDH: Subdural haemorrhage; TTP: Thrombotic thrombocytopaenic purpura; AFLP: Acute fatty liver of pregnancy.

## Competing interests

Author declares that he has no competing of interests. This is a self-funded work by the author.

## Authors’ contributions

MP carried out designing, analyzing and writing the report and drafting the final manuscript by himself.

## Authors’ information

Dr. Malitha Patabendige MBBS (Hons), Medical Officer, University Obstetrics Unit, De Soysa Hospital for Women, Colombo, Sri Lanka.
